# Expression profile of SYNE3 and bioinformatic analysis of its prognostic value and functions in tumors

**DOI:** 10.1186/s12967-020-02521-7

**Published:** 2020-09-18

**Authors:** Liwei Liao, Longshan Zhang, Mi Yang, Xiaoqing Wang, Weiqiang Huang, Xixi Wu, Hua Pan, Lu Yuan, Wenqi Huang, Yuting Wu, Jian Guan

**Affiliations:** 1 Department of Radiation Oncology, Nanfang Hospital, Southern Medical University, Guangzhou, Guangdong China; 2 Chronic Airways Diseases Laboratory, Department of Respiratory and Critical Care Medicine, Nanfang Hospital, Southern Medical University, Guangzhou, Guangdong China

**Keywords:** SYNE3, Expression profile, CeRNA network, Immune infiltration, Bioinformatic analysis, Tumor

## Abstract

**Background:**

Spectrin repeat containing nuclear envelope family member 3 (SYNE3) encodes an essential component of the linker of the cytoskeleton and nucleoskeleton (LINC) complex, namely nesprin-3. In a tumor, invasiveness and metastasis rely on the integrity of the LINC complex, while the role of SYNE3/nesprin-3 in cancer is rarely studied.

**Methods:**

Here, we explored the expression pattern, prognostic value, and related mechanisms of SYNE3 through both experimental and bioinformatic methods. We first detected SYNE3 in BALB/c mice, normal human tissues, and the paired tumor tissues, then used bioinformatics databases to verify our results. We further analyzed the prognostic value of SYNE3. Next, we predicted miRNA targeting SYNE3 and built a competing endogenous RNA (ceRNA) network and a transcriptional network by analyzing data from the cancer genome atlas (TCGA) database. Interacting genes of SYNE3 were predicted, and we further performed GO and KEGG enrichment analysis on these genes. Besides, the relationship between SYNE3 and immune infiltration was also investigated.

**Results:**

SYNE3 exhibited various expressions in different tissues, mainly located on nuclear and in cytoplasm sometimes. SYNE3 expression level had prognostic value in tumors, possibly by stabilizing nucleus, promoting tumor cells apoptosis, and altering tumor microenvironment. Additionally, we constructed a RP11-2B6.2-miR-149-5p-/RP11-67L2.2-miR-330-3p-SYNE3 ceRNA network and a SATB1-miR-149-5p-SYNE3 transcriptional network in lung adenocarcinoma to support the tumor-suppressing role of SYNE3.

**Conclusions:**

Our study explored novel anti-tumor functions and mechanisms of SYNE3, which might be useful for future cancer therapy.

## Background

Tumor malignancies has been greatly endangering public health for a long time. It was estimated that there were 17.0 million new cancer cases and 9.5 million cancer deaths worldwide in 2018. Therefore, prevention and treatment of tumor became a significant health-care issue.

With the development of sequencing technologies, genomic markers are increasingly used in tumor study, including tumor gene mutations and patterns of tumor gene expression. Gene markers can help with judging tumor development process, predicting treatment effect, reducing recurrence rate and mortality, and prolonging survival. Most cancer types have some classic but limited biomarkers which cannot always meet clinical requirements. Therefore, increasing studies are focusing on finding new and effective tumor gene markers in various cancer types [1–3]. However, to meet more complex and more personalized clinical needs, tumor-related genes still need further and deeper exploitation.

Spectrin repeat containing nuclear envelope family member 3 (SYNE3) encodes an crucial component of the linker of the cytoskeleton and nucleoskeleton (LINC) complex, namely nesprin-3 (nuclear envelope spectrin repeat protein 3) [4]. LINC complex is closely related to cancer progression [5]. A malignant tumor has unique biological characteristics such as abnormal cell differentiation and proliferation, loss of growth control, invasiveness, and metastasis, making tumors easy to recur, metastasize or spread. Moreover, all these processes need a morphological change of cells and genetic regulation, which rely highly on cytoskeleton remodeling and functions of nucleoskeleton. As LINC is vital for maintenance and regulation of the cytoskeleton and the nucleoskeleton, it is crucial in cancer development as well [6, 7]. Since nesprin-3/SYNE3 was initially discovered, it was mostly studied in stem cells. Nesprin-3 was reported to engage in nucleus maintaining [8], cell migration [9, 10] and cell differentiation [11–13]. Recent studies began to focus on its roles in cancer, suggesting that nesprin-3 mediated tumor cell migration in lung cancer [14] and fibrosarcoma cells [15]. Also, the copy number change of SYNE3 was significantly observed in various epithelial cancer types [16]. However, with present researches, our knowledge of nesprin-3/SYNE3 in tumor is still relatively limited.

In this study, we tried to have a fundamental analysis of SYNE3 in both experimental and bioinformatic aspects. Firstly, expression profile and histological distribution of SYNE3 in various BALB/c mice tissues, normal human tissues, and paired tumor tissues were displayed. For its potential roles in cancer, the prognostic value of SYNE3 was discussed in patients with tumors. Additionally, SYNE3 and its interacting genes were listed, and their functions were analyzed. At last, we investigated how SYNE3 expression affected tumor immune infiltration, thus influencing prognosis.

## Methods

### Specimen collection

We purchased five male 4-week-old BALB/c mice from the Experimental Animal Center of Southern Medical University and collected 12 types of normal tissues from each of them, including tissues from gut, lung, liver, thyroid, brain, spleen, trachea, kidney, esophagus, stomach, heart and pancreas. The investigation conforms with the Guide for the Care and Use of Laboratory Animals published by the US National Institutes of Health (NIH Publication No. 85–23, revised 1996).

We also collected nine types of specimen of resected tumor tissues and paired normal tissues from 72 patients with cancer undergoing surgical resection in Nanfang hospital during 2019–1 to 2019–10. The tumor tissues included 11 cases of liver, 5 cases of cervix, 10 cases of colon, 3 cases of small intestine, 8 cases of kidney, 4 cases of esophagus, 11 cases of breast, 10 cases of lung squamous cell carcinoma (LUSC) and 10 cases of lung adenocarcinoma (LUAD). Informed consent was obtained from each patient on the day of admission. The study protocol conforms to the ethical guidelines of the World Medical Association, Declaration of Helsinki Ethical Principles for Medical Research Involving Human Subjects adopted by the 18th WMA General Assembly, Helsinki, Finland, June 1964, as revised in Tokyo 2004. All patients have received no anti-tumor treatments before their surgeries, including radiotherapy, chemotherapy, biological immunotherapy, and multiple operations. Over 80% of tumor cells were contained in each cancer specimen, as certified by microscopic observation.

### Immunohistochemistry (IHC)

Collected tissues were fixed in 4% paraformaldehyde, dehydrated, then embedded in paraffin and sliced into sections of 4 μm thickness. The sections were baked in the oven at 65 °C for 1.5 h and then hydrated. Next, antigen retrieval was practiced in boiling Tris/EDTA buffer (pH 9.0) at 100 °C for 12 min, then followed by incubation in 3% superoxol for 15 min to block endogenous peroxidase. The tissues were incubated with anti-SYNE3 monoclonal antibody diluted 25-fold (Thermo Fisher Scientific, USA) at 4 °C overnight. Then anti-rabbit secondary antibody (ORIGENE) was added and incubated at room temperature for 1 h. Next, diaminobenzidine (DAB) was used to reveal the color of antibody staining. Finally, the slides were mounted and observed under an optical microscope (Leica, Germany). The expression of SYNE3 was calculated concerning both the positivity proportion of stained tumour cells and the staining intensity. The positivity proportion was scored as “0”, 0%; “1”, 1–25%; “2”, 26–50%; “3”, 51–75% and “4”, > 75%. The staining intensity was scored as “0” (no staining), “1” (weakly stained), “2” (moderately stained) and “3” (strongly stained). Both percent positivity of cells and staining intensity were evaluated in a double blinded manner. The staining of SYNE3 was assessed as follow: a final staining score of < 3 was regarded as “none”; a final staining score of 3 was regarded as “weak”; a final staining score of 4 was assessed as “middle” and a final staining score of ≥ 5 was concerned as “strong”.

### Bioinformatics mining of SYNE3

We acquired the chromosome localization of SYNE3 on the GeneCards database (https://www.genecards.org/). SYNE3 gene structure was analyzed on the Ensembl database (http://asia.ensembl.org/), with its protein structure analyzed in the Uniprot database (https://www.uniprot.org/), then visualized by using Illustrator for biological sequences software (IBS, http://ibs.biocuckoo.org/). We further accessed transcripts of the SYNE family from Uniprot and constructed a phylogenetic tree by using MAFFT (https://mafft.cbrc.jp/alignment/server/) and ITOL (https://itol.embl.de/gallery.cgi). The protein-sequence comparison among different species was analyzed by utilizing DNAMAN software (lynnonBiosoft, USA).

Transcriptome data of diverse tumor tissues and their precancerous tissue were downloaded from The Cancer Genome Atlas (TCGA) (https://portal.gdc.cancer.gov/) database. Expression results in normal tissue were analyzed by utilizing the University of California, Santa Cruz (UCSC) Xena browser (https://xenabrowser.net). Expression pattern, Disease-free survival (DFS) analysis and overall survival (OS) analysis of SYNE3 in 33 cancer types were performed on gene expression profiling and interactive analyses (GEPIA) database (http://gepia.cancer-pku.cn/).

MiRNAs targeting SYNE3 were predicted based on four different databases (DIANA-TarBase v8, miRWalk, TargetScanHuman and mirDIP). Furthermore, lncRNA-miRNA relationships of predicted SYNE3-associated miRNAs were obtained by overlapping results from starBase (http://starbase.sysu.edu.cn/) and DIANA-LncBase v2 (http://carolina.imis.athena-innovation.gr/diana_tools/web/index.php?r=lncbasev2%2Findex-predicted). The overlapping result was pictured by website Bioinformatics & Evolutionary Genomics (http://bioinformatics.psb.ugent.be/webtools/Venn/). Finally, a competitive endogenous RNA (ceRNA) network regulating SYNE3 was constructed by using Cytoscape (version 3.6.0, http://www.cytoscape.org/).

We screened interacting genes of SYNE3 from STRING (Search Tool for the Retrieval of Interacting Genes) database (http://string-db.org) with a confidence score of ≥ 0.4 was eligible for protein–protein interactions network (PPI) network construction and used Cytoscape to adjust it.

We performed gene ontology (GO) enrichment analysis on 41 interactive genes of SYNE3 by online Database for Annotation, Visualization and Integrated Discovery (DAVID; https://david.ncifcrf.gov/summary.jsp), and then the results of biological process (BP), cellular component (CC), molecular function (MF) were accessed to explore their functions. Moreover, Kyoto Encyclopedia of Genes and Genomes (KEGG) pathway of those 41 genes was analyzed in KOBAS 3.0 (http://kobas.cbi.pku.edu.cn/). Then, website imageGP (http://www.ehbio.com/ImageGP/) was utilized to make GO enrichment plot and KEGG plot. Simultaneously, the most enriched pathway was visualized by using KEGG Mapper, a collection of tools for KEGG mapping, with enriched genes marked in orange.

Tumor IMmune Estimation Resource (TIMER; https://cistrome.shinyapps.io/timer/) database was used to investigate how SYNE3 expression influenced tumor microenvironment, and the connection of different immune infiltration levels and corresponding prognosis. Gene Set Enrichment Analysis (GSEA) was performed using software GSEA v4.0.3 Java Web Start using gene set C7: immunologic signatures gene sets acquired from Molecular Signatures Database (MsigDB). Moreover, the correlation between SYNE3 and immune-cell markers was analyzed by GEPIA.

### Statistical analysis

All our data were analyzed on software GraphPad Prism (version 6.02, San Diego, California, USA). We applied Student’s *t* test to compare two different groups and used the Pearson correlation method for correlation analysis of SYNE3. P < 0.05 was considered statistically significant and false discover rate (FDR) < 0.05 was regarded statistically credible. The strength of the correlation was determined using the following guide for the correlative value: 0.00–0.19 “not related,” 0.20–0.39 “weak,” 0.40–0.59 “moderate,” 0.60–0.79 “strong,” 0.80–1.0 “very strong.”

## Results

### Structure and dendrogram of SYNE3

**Fig. 1:**
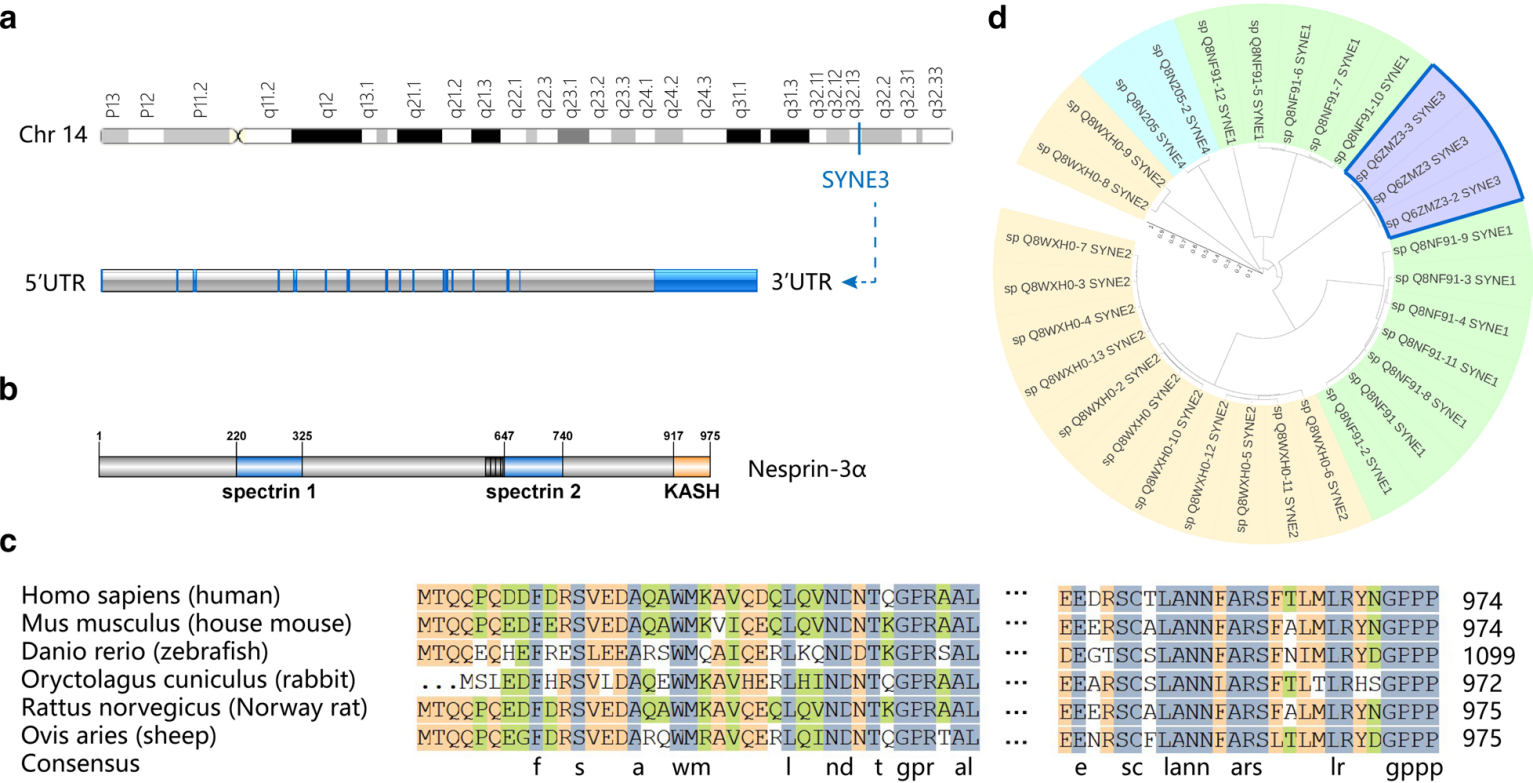
Gene structure, protein structure and conservative analysis of SYNE3. **a** Chromosome localization and gene structure of SYNE3 in human. **b** Structure of nesprin-3ɑ, the most common SYNE3 encoding protein. **c** Comparison of protein sequences encoded by SYNE3 among six different species. **d** Phylogenetic tree of SYNE family

SYNE3 located at 14q32.13, containing 18 exons and 17 introns (Fig. 1a). SYNE3 encoded 2 isoforms, nesprin-3α and nesprin-3β. Nesprin-3α was the dominant isoform and the main undertaker of nesprin-3 functions. The protein structure of nesprin-3α consisted of two parts of spectrin repeats, a KASH domain and a coiled-coil region (Fig. 1b). To study the conservation of SYNE3 among distinct species, we compared protein sequences encoded by SYNE3 among six different species (Fig. 1c), showing that Homo sapiens SYNE3 shared 77, 30, 78, 77 and 79% identity to Mus musculus, Danio rerio (zebrafish), Oryctolagus cuniculus, Rattus norvegicus and Ovis aries, respectively. It presented that SYNE3 was highly conserved in mammals, but varied significantly between human and zebrafish, a common animal model to study SYNE3 functions in neurons and stem cells. Moreover, a phylogenetic tree was constructed to analyze the conservative relationship among SYNE family members (Fig. 1d). In this tree, the SYNE family was divided into two main clusters; one included two SYNE2 isoforms, and the other contained the other SYNEs. More specifically, SYNE3, SYNE1, and the left SYNE2 isoforms were in the same subset, while SYNE4 was not.

### Expression of SYNE3 in BALB/c mice tissues

**Fig. 2:**
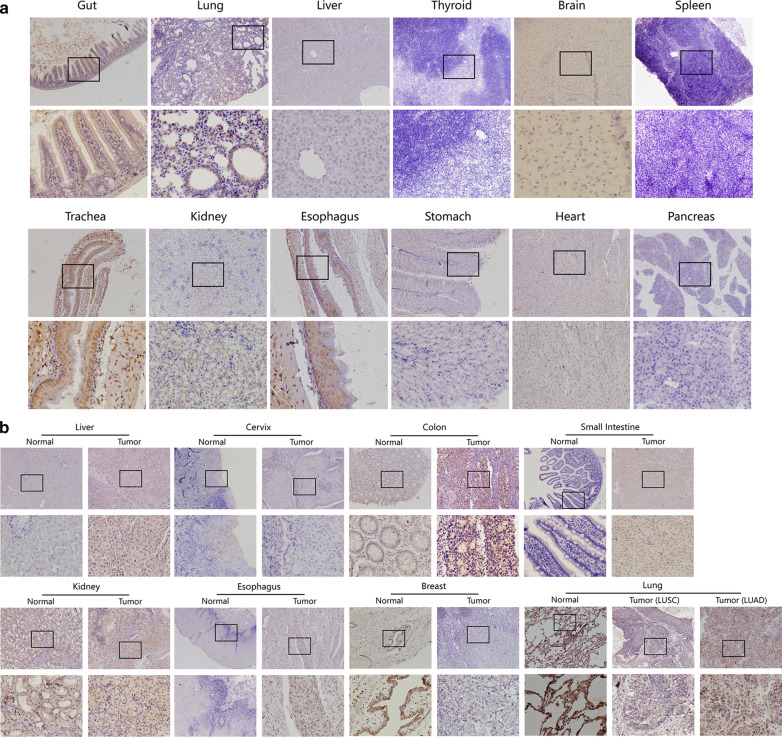
Expression pattern of SYNE3 in BALB/c mice and human. **a** distribution of SYNE3 in tissues from BALB/c mice (100×; 400×; five samples in each group). **b** Immunohistochemistry results of SYNE3 performed in both normal and tumor tissues of human (100×; 400×; more than three samples in each group)

As BALB/c mice was a widely-used animal model in studies on tumorigenesis and tumor migration, we first detected the SYNE3 expression of various normal tissues in BALB/c mice (Fig. 2a). SYNE3 staining was positive in the gut, lung, trachea, esophagus, stomach, and heart, while not obvious in the liver, thyroid, brain, spleen, kidney, and pancreas. In the gut, weak positive staining of SYNE3 was found in the cytoplasm of the intestinal villi and gland. The lung presented moderate staining in the nucleus of epithelial cells. SYNE3 was strongly detected in the nucleus in all layers of the trachea, and moderate in the cytoplasm in the mucosal layer. For the esophagus, nucleus staining was strong in all layers, and cytoplasmic staining was moderate in all layers except submucosal cells. In the stomach, weak staining was observed in the cytoplasm of chief cells and parietal cells. In the heart, only the nucleus of cardiomyocytes exhibited weak staining.

### Expression of SYNE3 in normal tissues and tumor tissues of the human

We carried out IHC on samples of eight types of normal human tissues and their corresponding tumor tissues (Fig. 2b). In liver, only the cancer cells presented weak SYNE3 staining in the cytoplasm. No SYNE3 was detected in both the normal and tumor cervix tissues. Weak positive staining was found in the cytoplasm of normal gut epithelial cells, compared with moderate positive staining in cytoplasm of colon adenocarcinoma cells. Both small intestine and esophagus showed no staining in normal tissue and weak staining in their cancer cells. In terms of the kidney, moderate to strong positive staining was present in both the nucleus and cytoplasm of normal renal tubular epithelial cells, while only weak staining was found in whole cells of renal clear cell carcinoma. Breast and lung presented the most obvious expression differences between the normal and the tumor tissues. In normal tissues, strong positive staining was observed in the nucleus and cytoplasm of breast luminal epithelial cells and the nucleus of lung alveolar epithelial cells. While in the corresponding tumor samples, SYNE3 staining was only weakly present in cancer cells.

We also referred to UCSC database (Additional file 1: Fig. S1a). Compared with our experimental data, SYNE3 mRNA was expressed mostly in the breast and lung as well. It was contradictory that the SYNE3 mRNA level in the kidney was low, though our IHC result showed moderate to strong positive staining.

Meanwhile, according to GEPIA database, SYNE3 was significantly expressed less in 9 cancer types, including invasive breast carcinoma (BRCA), lung adenocarcinoma (LUAD), and lung squamous cell carcinoma (LUSC)(P < 0.0001), which were consistent with our IHC results in these tumors. However, only in acute myeloid leukemia (LAML), SYNE3 expression level is higher in tumor tissue compared with normal one (P < 0.0001). The expression change in LAML is the most noticeable, with its fold change reaching 9.26 (Additional file 1: Fig. S1b).

### Prognostic value of SYNE3

To discover prognostic cancer value of SYNE3, we investigated the connection between SYNE3 expression level and DFS of patients with cancer, finding that higher SYNE3 expression brought longer lifespan to patients with renal clear cell carcinoma (KIRC) (P = 0.033, HR = 0.67) or with cervical squamous cell carcinoma and endocervical adenocarcinoma (CESC) (P = 0.046, HR = 0.56) (Additional file 2: Fig. S2a). In terms of analysis of OS, five out of 33 kinds of the tumor showed significant results (Additional file 2: Fig. S2b). Specifically, we found that in KIRC (P < 0.001, HR = 0.58, Kaplan–Meier), LUAD (P = 0.011, HR = 0.67), CESC (P = 0.02, HR = 0.57) and squamous cell carcinoma of head and neck (HNSC) (P = 0.046, HR = 0.76), patients with high SYNE3 expression survive longer than others with low SYNE3 expression level. However, the case was in reverse in brain lower-grade glioma (LGG) (P = 0.034, HR = 1.5), in which patients with higher SYNE3 expression live even shorter. These results suggested SYNE3 prognostic value in various cancer types.

### Construction of ceRNA network of SYNE3 in LUSC

**Fig. 3:**
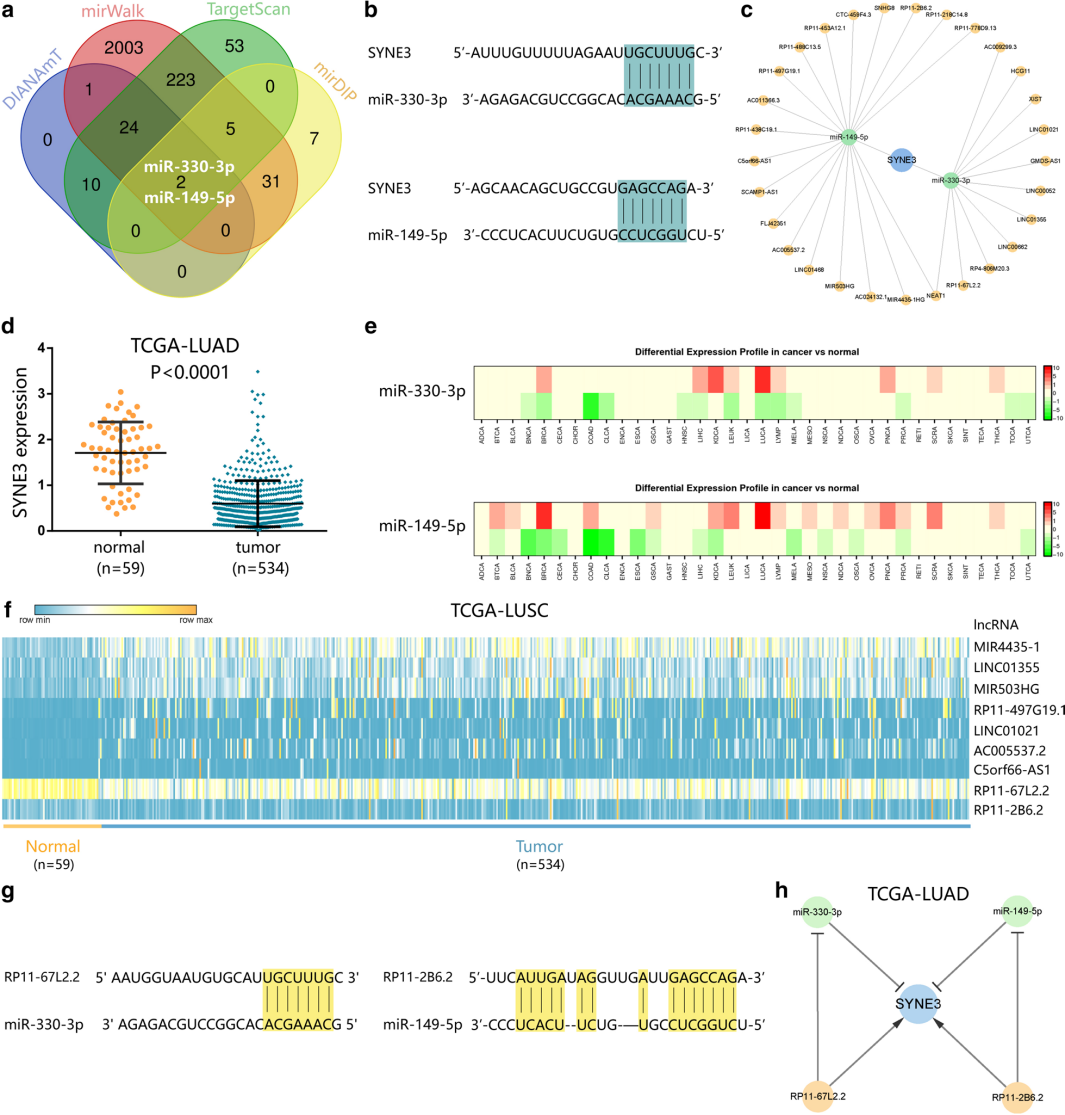
Networks regulating SYNE3. **a** Result of predicted miRNAs using 4 different database. Hsa-miR-330-3p and hsa-miR-149-5p were finally screened by overlapping these data and the result was visualized on Bioinformatics & Evolutionary Genomics website. **b** Predicted interaction between miR-149-5p/miR-330-3p and SYNE3. **c** Construction of ceRNA network of SYNE3. **d** SYNE3 expression in LUAD according to TCGA database. **e** Differential expression profile in cancer vs normal of miR-330-3p and miR-149-5p. **f** LncRNAs in the network differently expressed in normal vs tumor in LUAD based on TCGA database. **g** Predicted interaction of miR-149-5p–RP11-2B6.2 and miR-330-3p–RP11-67L2.2. **h** A ceRNA network in LUAD composed of SYNE3, miR-149-5p, miR-330-3p, RP11-2B6.2 and RP11-67L2.2

We analyzed the upstream regulation of SYNE3, screening miRNAs or lncRNAs, which targeted SYNE3. We found 37 miRNAs possibly targeting SYNE3 from DIANAmT database, 2289 from miRWalk database, 317 from TargetScan database, and 45 from mirDIP database (Fig. 3a). As a result, we screened hsa-miR-330-3p and hsa-miR-149-5p as the most vital miRNA regulators by overlapping predictions of four databases. We also presented the complementary sequences between SYNE3 and miR-330-3p and miR-149-5p (Fig. 3b). Then, we predicted eleven lncRNAs that can bind with hsa-miR-330-3p and 19 lncRNAs targeting miR-149-5p. In this way, we constructed a lncRNA-miRNA-mRNA network (Fig. 3c), in which lncRNA competitively bound with miRNA and weakened the suppression from miRNA to SYNE3.

The regulatory mechanisms we predicted were only limited to the genetic level for using TCGA data, as there should be many mechanisms after transcription that can also influence the gene expression. Therefore, to better verify our predicted mechanisms, we tend to choose a cancer tissue whose mRNA difference is consistent with protein difference to minimize influence of modifications after transcription. Based on our results in IHC and bioinformatic analysis, BRCA, LUSC and LUAD presented most significant differences in both mRNA and protein level of SYNE3 between normal and tumor tissues. As previous studied have revealed SYNE3 connection with lung cancer development [14, 16] compared with few reports on BRCA, so we chose to focus more on SYNE3 role in lung cancer in this article. Considering that SYNE3 expression difference can lead to OS difference in LUAD, we used LUAD as an example to illustrate our ceRNA network here.

SYNE3 expression (Fig. 3d), miRNAs expression (Fig. 3e) and expression of lncRNAs were compared between tumor tissues of LUAD and normal ones. SYNE3 expressed significantly lower in tumor tissues of LUAD, and both two miRNAs expressed higher in tumor tissues instead. In terms of lncRNAs, RP11-2B6.2 and RP11-67L2.2 presented a significant decrease in their expression (Fig. 3f). Combining with these analyses and interactions predicted by the database above (Fig. 3g), we might suppose that RP11-2B6.2 and RP11-67L2.2 bind with hsa-miR-149-5p and hsa-miR-330-3p respectively to weaken miRNA suppression toward SYNE3 in LUAD (Fig. 3h).

### Transcriptome analysis of SYNE3 in LUSC

**Fig. 4:**
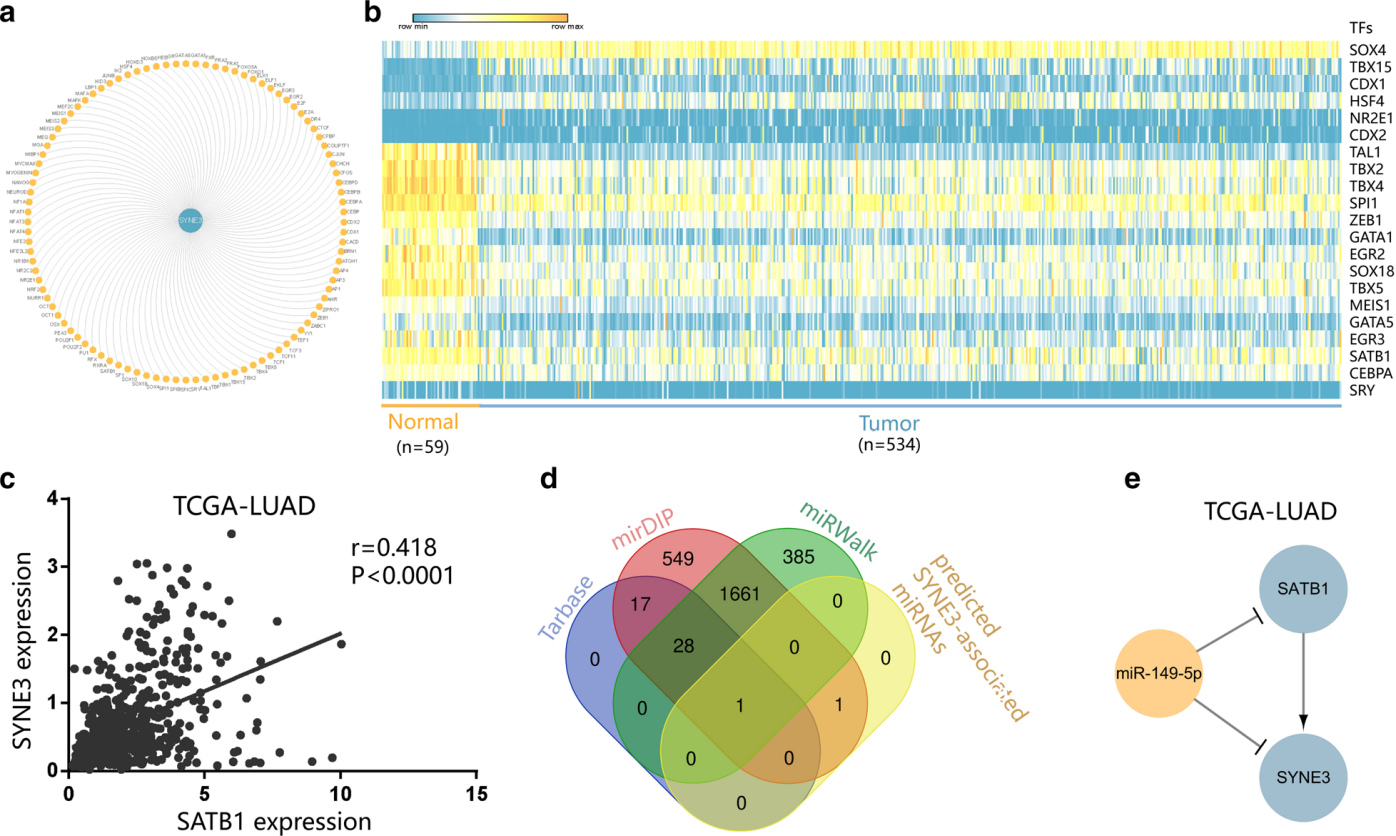
Transcriptional network of SYNE3. **a** predicted SYNE3-associated transcriptional factors based on the results of GCBI online database. **b** TFs differently expressed in normal tissues and tumor tissues of LUAD. **c** Pearson correlation coefficient between SYNE3 expression and SATB1 expression. **d** MiRNA screened to regulate SATB1, using the data of Tarbase, mirDIP, miRWalk and previous analysis. **e** transcriptional network among SYNE3, miR-149-5p and SATB1 in LUAD

To better understand the upstream mechanisms of SYNE3, we continued to do some transcriptome analysis on SYNE3, still using the example of LUAD. First, we constructed a transcriptional regulatory network involving 100 transcriptional factors predicted by GCBI database (Fig. 4a). Then, we selected 21 TFs with significant expression differences in normal tissues and tumor tissues of LUAD (Fig. 4b). Among them, SATB1 was correlated with SYNE3 expression (Fig. 4c, r = 0.418, P < 0.0001). Combined with the analysis of ceRNA network above, we wondered if the predicted SYNE3-related miRNAs also participated in TF regulation. By using the data of Tarbase, mirDIP, miRWalk and our predicted results, we identified miR-149-5p as an upstream regulator of SATB1 (Fig. 4d). Moreover, in LUAD, SATB1 and SYNE3 were both downregulated, while miR-149-5p was expressed more. Based on these, a SATB1-miR-149-5p-SYNE3 transcriptional network in LUAD was constructed (Fig. 4e).

### KEGG and GO pathway enrichment analysis of SYNE3

**Fig. 5:**
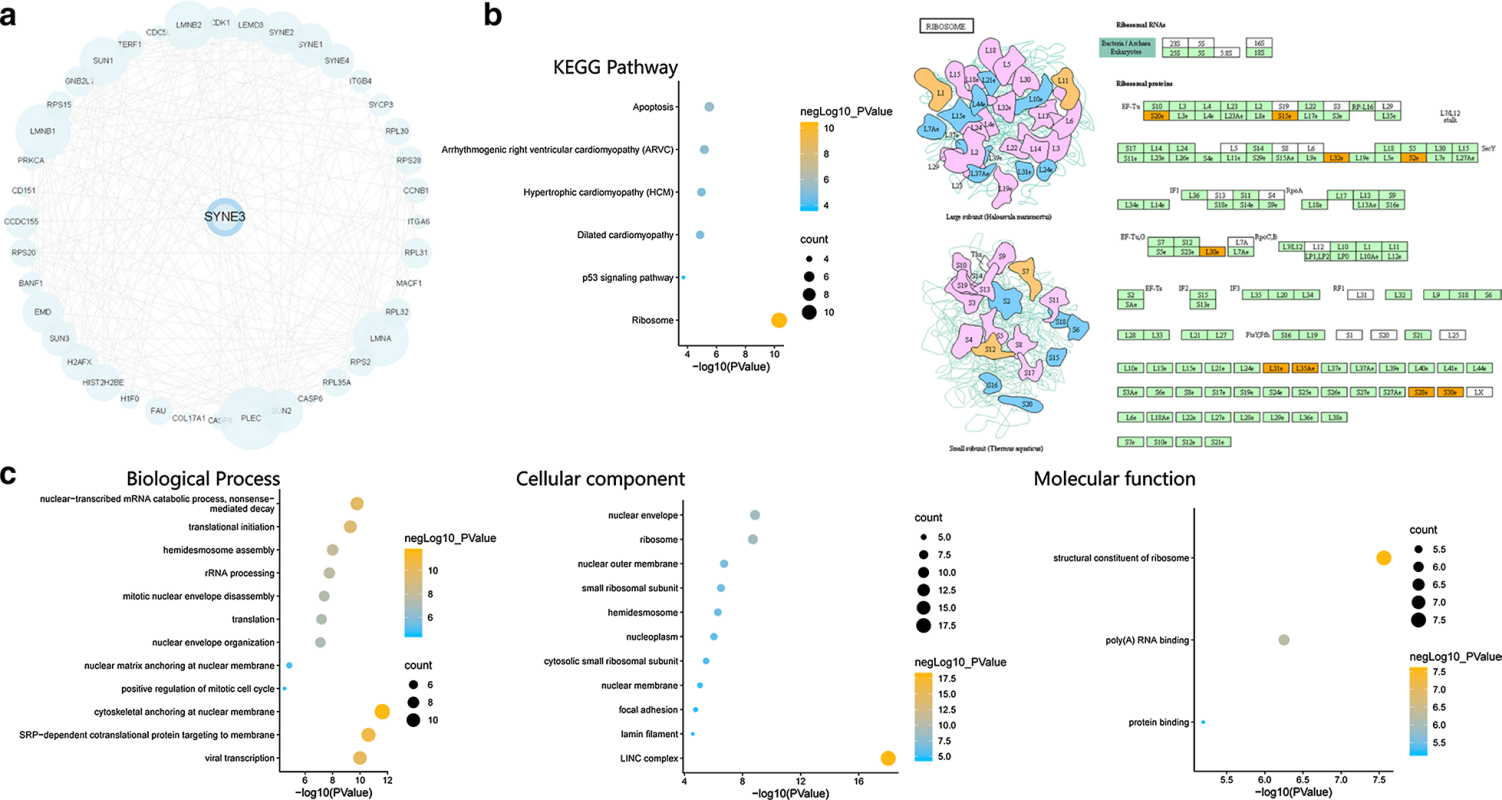
Kyoto Encyclopedia of Genes and Genomes (KEGG) and Gene Ontology (GO) enrichment analysis of SYNE3. **a** an protein–protein interacting network of SYNE3 constructed with data from STRING. **b** KEGG enrichment was performed by using KOBAS 3.0 database, and the most enriched pathway map was pictured based on map from KEGG Mapper. **c** GO analysis was performed with DAVID (Database for Annotation, Visualization and Integrated Discovery) in three aspects: Molecular Function, Biological Process and Cellular Component

To investigate the downstream regulation of SYNE3, a PPI network (Fig. 5a) was built with 40 interacting genes of SYNE3 predicted by the STRING database. We performed KEGG analysis of SYNE3 and its 40 interacting genes on KOBAS. Finally, We acquired 6 KEGG pathways these 41 genes were enriched in, specifically ribosome, apoptosis, arrhythmogenic right ventricular cardiomyopathy (ARVC), hypertrophic cardiomyopathy (HCM), dilated cardiomyopathy and p53 signaling pathway, with KEGG map of ribosome especially presented (Fig. 5b).

For GO enrichment analysis, SYNE3 and its interacting genes were significantly and credibly enriched in 12 Biological processes (BPs), 11 Cellular components (CCs), and 3 Molecular functions (MFs) (Fig. 5c). These genes mainly encoded protein from LINC, cytoskeleton, nucleoskeleton and ribosome, likely participating in nucleus adjustment and transcription.

### Immune infiltration level analysis in cancer associated with SYNE3

Higher SYNE3 expression was linked to better clinical outcomes in our analysis, suggesting its tumor-suppressing functions. Here, we explored this function in the aspect of immunity. First, we screened cancer types whose immune infiltration was correlated with SYNE3 expression. Accordingly, we found that SYNE3 expression was significantly correlated with dendritic cell, neutrophil, CD4 + T cell, macrophage, CD8 + T cell and B cell in 30, 30, 27, 26, 25, 25 and 22 types of cancer respectively.

**Fig. 6:**
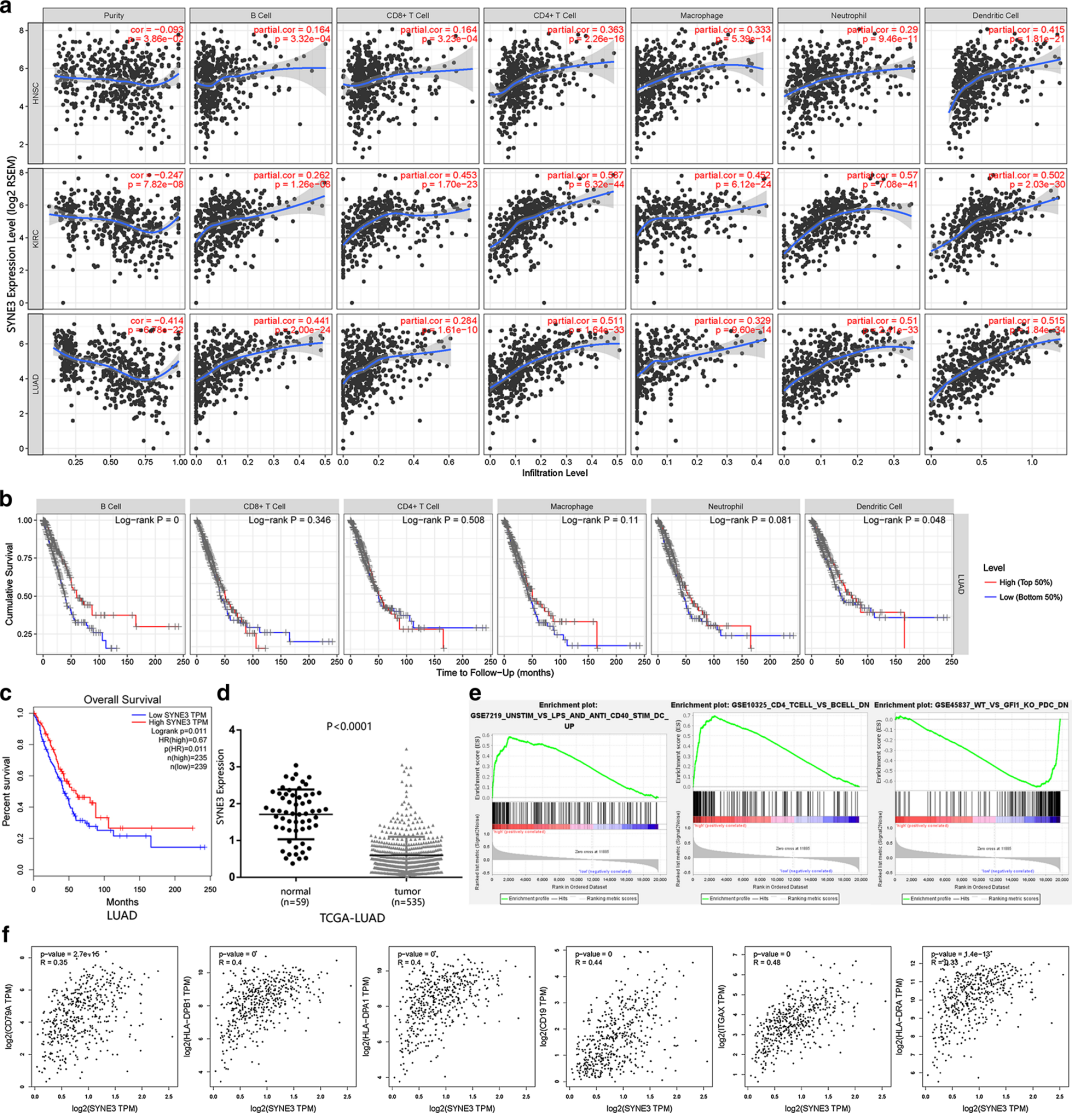
The role SYNE3 played in tumor microenvironment. **a** Immune infiltration level associated with SYNE3 in HNSC, KIRC and LUAD analyzed in TIMER. **b** The relationship between immune cell infiltration and lung adenocarcinoma prognosis, higher infiltration level of B cell and dentritic cell were significantly associated with better clinical outcome of LUAD. **c** Higher SYNE3 expression was correlated with longer OS of patients with lung adenocarcinoma. **d** SYNE3 expression was significantly downregulated in LUAD compared with that in normal lung tissues. **e** GSEA analysis grouped by SYNE3 expression level. **f** The correlation between SYNE3 expression and expression of B cell and dentritic cell based on GEPIA database

We then analyzed each cancer type and found ten types of cancer with significant results in all immune cell types and purity. Mostly, in HNSC, KIRC and LUAD, both OS and immune infiltration were positively correlative with SYNE3 expression (Fig. 6a). Among these three cancer types, the survival of LUAD presented a closer relationship with the level of immune cell infiltration (Fig. 6b), which was positively correlated with the infiltration of B cell and dendritic cell.

Hence, we tended to figure out the role of SYNE3 expression in the immune infiltration of LUAD. In LUAD, higher SYNE3 expression corresponded with better clinical outcome (Fig. 6c), and the expression of SYNE3 was significantly downregulated (Fig. 6d) overall. We performed GSEA analysis on 535 TCGA samples divided into a high group (267 samples) and a low group (268 samples) by expression level. The result revealed that pathways indicative of DC cell and B cell activation significantly correlated with SYNE3 expression (Fig. 6e). To further confirm this point, we also analyzed the relationship between SYNE3 and biomarkers of DC cell [17, 18] and B cell [17] (Fig. 6f), with all correlations significant.

## Discussion

As a linker protein of cytoskeleton and nucleoskeleton, SYNE3/nesprin-3 has been shown to play a vital role in 3D cell migration [19] and tumor cell movement [15] in previous studies. However, these researches too limited to reveal SYNE3 functions in the tumor. Therefore, in our study, we hoped to provide some basic knowledge of SYNE3 and analyze its roles in cancer.

Based on our analysis, SYNE3 was conserved among mammals and its family members, suggesting some vital roles of SYNE3 to keep through evolution. Moreover, SYNE3 shared more similarities with SYNE1 and SYNE2 than SYNE4 in its structure and may in its functions as well.

We investigated SYNE3 expression in various tissues. In normal human tissues, SYNE3 was strongly detected in breast and lung, corresponding with the highest mRNA level in these two tissues. Moreover, the expression difference between normal and tumor tissues was most obvious in breast and lung as well in both IHC and GEPIA. However, conflicts also existed; for instance, the kidney was of low mRNA but moderate to high SYNE3 staining. On the one hand, our tissues were just randomly selected cases, so they cannot represent general conditions like data from UCSC or GEPIA. On the other hand, the mRNA level was not always consistent with protein level, and this discrepancy is also common in studies on other genes [20, 21], though there was no related study on SYNE3 expression. Also, though SYNE3 had been reported to exist widely, its expression was not always high enough to be detected.

In our predictive analysis, we found a higher SYNE3 expression level associated with longer OS in patients with KIRC, LUAD, CESC or HNSC except for LGG with a negative correlation. In most cases, the expression of SYNE3 was also downregulated in tumor, indicating its anti-cancer roles in these cancer types, especially in LUAD and CESC. In KIRC, though SYNE3 expression showed no difference in KIRC, our IHC result detected decreased expression of SYNE3. We suppose post-transcriptional regulation and translation regulation might be responsible for this phenomenon as TCGA data mainly focused on transcriptional level. A recent study showed that knockdown of SYNE3 led to DNA damage, genome organization loss, and transcriptional changes [22], and these events were closely related to tumorigenesis for this mechanism had been reported in SYNE1 [23]. Therefore, SYNE3 participated in some anti-tumor events, and there might be various mechanisms to adjust SYNE3 expression. However, it was noted that SYNE3 unusually roles as a promoting factor in LGG. SYNE3 presented no difference in transcriptional level between tumor tissues and normal tissues of LGG based on TCGA data, but it was unclear if there was a SYNE3 expression discrepancy in protein level for lack of specimens. Moreover, the mechanisms by which genes affect tumor prognosis are extremely complex. A gene can not only directly affect the biological characteristics of tumor cells, but can also remodel tumor microenvironment to influence tumor outcome. So this phenomenon might suggest some special functions of SYNE3 in LGG, which needs further study.

CeRNA is a conception firstly proposed in 2011 [24]. In ceRNA network, non-coding RNA, likely lncRNA or circRNA, can competitively bind with miRNA, thereby weaken the repression from miRNA to mRNA. We identified hsa-miR-330-3p and hsa-miR-149-5p as key regulatory miRNAs of SYNE3. MiR-330-3p was a recognized cancer-promoting factor. It was reported that miR-330-3p could target the gene of program cell death 4 (PDCD4) to promote tumors [25]. MiR-330-3p is also related to metastasis and invasion of lung cancer, and glutamate receptor 3 (GRIA3) [26] and human manganese superoxide dismutase 2 (hSOD2) [27] are its two target genes. Researches found miR-330-3p is correlated with bad prognosis in breast cancer as well [28], in which miR-330-3p can target Collagen and Calcium Binding EGF Domains 1 (CCBE1) to promote cancer metastasis [29]. However, the function of miR-149-5p depends on specific tumor types. Higher miR-149-5p led to lower OS in renal cell carcinoma (RCC), though miR-149-5P also presented some tumor-suppressing functions in RCC [30], suggesting that further studies were needed to explain this paradox. MiR-149-5p can help to disable the invasion and proliferation of medullary thyroid carcinoma cells [31] and suppress the development of non-small cell lung cancer [32], while its role seems to be controversial in hepatocellular carcinoma [33, 34]. Combined with our survival analysis, tumor-promoting functions of miRNAs displayed more obvious in KIRC, CESC, LUAD and HNSC. While for LGG, there might be other mechanisms related to its heterogeneity [35], unique progressive features or micro-environment, causing a contrary result.

In our ceRNA network, NEAT1 is a tumor suppressors targeting both miR-330-3p and miR-149-5p. NEAT1 can be mediated by Tumor protein P53 (P53), a critical tumor suppressor, to suppress transformation and cancer initiation [36]. Meanwhile, only a few of the rest lncRNAs have been reported, including both tumor suppressors and tumor enhancers. These results showed double regulations in this ceRNA network of SYNE3, and tumor-suppressing lncRNAs seem to role more dominantly. Moreover, a RP11-2B6.2-miR-149-5p-/RP11-67L2.2-miR-330-3p-SYNE3 ceRNA network and a SATB1-miR-149-5p-SYNE3 transcriptional network in LUAD. RP11-2B6.2 was identified as a positive regulator of IFN-I signaling pathway in Lupus nephritis [37] while there has been no report on RP11-67L2.2. Additionally, loss of SATB1 was proved to be a sign of poor clinical outcome in lung cancer [38]. In our study, SYNE3 presented lower expression in both mRNA and protein level in tumor tissues compared with that in normal tissues. Besides, SYNE3 expression level can influence the OS and immune microenvironment of LUAD, making our predicted networks are valuable for further study of SYNE3 in LUAD.

As for the PPI network of SYNE3, it was reasonable for SYNE3 to interact with Sad1 And UNC84 Domain Containing (SUN) family, a set of cytoskeleton linker proteins and the other SYNE members. The function of SYNE3 is correlated with these binding partners, which was supported by GO and KEGG analysis. Based on our analysis, SYNE3 is correlated with some tumorous processes. P53 and three interacting genes of SYNE3 were involved in the P53 signaling pathway, including cyclin-dependent kinase 1 (CDK1), Caspase 8 (CASP8) and cyclin B1 (CCNB1). CDK1 can adjust the cell cycle [39], CASP8 is referred to as an apoptosis regulator [40], and CCNB1 is vital for mitosis and proliferation [41]. Moreover, apoptosis, a pathway closely to P53, also enriched 5 SYNE3 interacting genes, involving CASP8 again, LMNA, CASP6, LMNB1 and LMNB2. Among them, downregulation of CASP6 induced suppression of the apoptosis of chronic myeloid leukemia cells [42]. LMNB1 and LMNB2 are coding genes for lamin B1 and lamin B2, respectively, which are nucleoskeleton components associated with cancer and aging [43]. Besides, nine interacting genes of SYNE3 code ribosomal proteins, indicating a possible association with transcription. Based on these, SYNE3 may suppress cancer by promoting tumor apoptosis, which can be realized through adjustment of the cell cycle, recruitment of immune cells, and alteration of nucleoskeleton and ribosome.

We finally focused on SYNE3 and the immune property of the tumor microenvironment. SYNE3 showed a good correlation with tumor immune infiltration, especially in LUAD, as its OS was most relevant with its tumor microenvironment, especially with B cell and DC cell. It has already been reported that B-cell infiltration correlated with a good prognosis in LUAD [44]. A dendritic cell is responsible for antigen presentation to T- and B-cells and activation natural killer (NK) cells and can activate anti-tumor immunity [45]. Therefore, regulating the infiltration of B cell and DC cell can be a significant pathway for SYNE3 to influence the survival of patients with LUAD. GSEA analysis also showed that higher SYNE3 expression relating to pathways enhancing B cell and DC cell. Furthermore, SYNE3 also significantly correlated with markers of B cell and DC cell. Therefore, we supposed that higher expression of SYNE3 helped with the production and collection of B cell and DC cell so that SYNE3 altered tumor microenvironment and presented significant and reasonable prognostic value in LUAD.

## Conclusions

Overall, our study firstly provided an overview of SYNE3 expression in diverse normal and tumor tissues. Then, we tended to explore the prognostic value of SYNE3. Though previous researches revealed its role in mediating tumor metastasis, we found higher SYNE3 expression correlated with even better clinical outcomes. We then analyzed tumor-suppressing functions of SYNE3, showing that SYNE3 might engage in apoptosis mediation of tumor cells. Moreover, SYNE3 expression can impact immune cell infiltration as well, adjusting the local anti-tumor immunity to be more active and effective. Our study revealed the unexpected anti-cancer functions of SYNE3, and provided a new perspective of SYNE family in tumors, though still lacking experimental verification. In addition to tumor metastasis, the role of SYNE3 in apoptosis, maintenance of nuclear stability, and tumor microenvironment regulation in cancer are more prominent, especially in lung adenocarcinoma. These findings might suggest a potential biomarker for tumor prognosis and therapy.

## Supplementary information

**Additional file 1: Figure S1.** Expression pattern of SYNE3 refer to database. **a** Expression profile of SYNE3 in normal tissues acquired from University of California, Santa Cruz (UCSC) database. **b** SYNE3 RNA Expression in both normal and tumor tissues of human accessed from Gene Expression Profiling Interaction Analysis (GEPIA) database.

**Additional file 2: Figure S2.** Prognostic analysis of SYNE3. **a** Two significant results (KIRC and CESC) of disease free survival analysis on SYNE3, performed on GEPIA. **b** Five significant results (KIRC, LUAD, CESC, HNSC and LGG) of disease free survival analysis on SYNE3, performed on GEPIA.

## Data Availability

All the analysis data were accessed from TCGA database (https://portal.gdc.cancer.gov/).

## Abbreviations

SYNE3: Spectrin repeat containing nuclear envelope family member 3
CeRNA: Competing endogenous RNA
TCGA: The cancer genome atlas
IHC: Immunohistochemistry
DFS: Disease-free survival
OS: Overall survival
GO: Gene ontology
BP: Biological process
CC: Cellular component
MF: Molecular function
KEGG: Kyoto Encyclopedia of Genes and Genomes
GSEA: Gene set enrichment analysis
FDR: False discover rate
LUAD: Lung adenocarcinoma
LUSC: Lung squamous cell carcinoma
DC: Dendritic
