# Supplementary figures and images for: Expression profile of SYNE3 and bioinformatic analysis of its prognostic value and functions in tumors
Source: J Transl Med. 2020 Sep 18;18:355. doi: 10.1186/s12967-020-02521-7 (PMC7501639; doi:10.1186/s12967-020-02521-7)

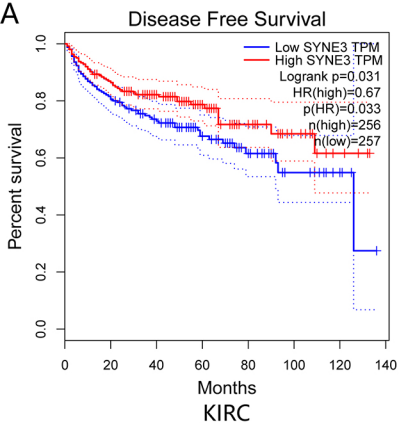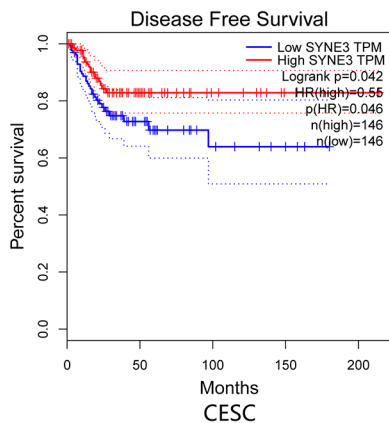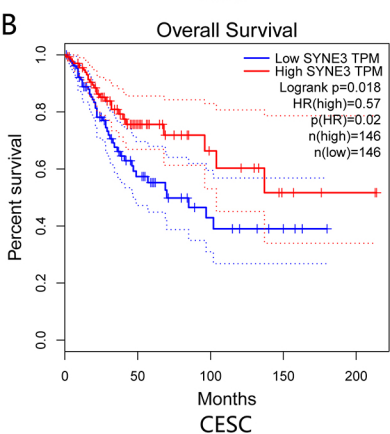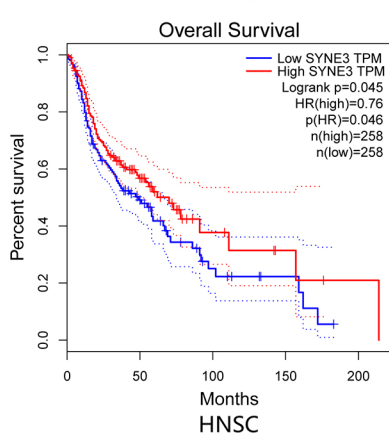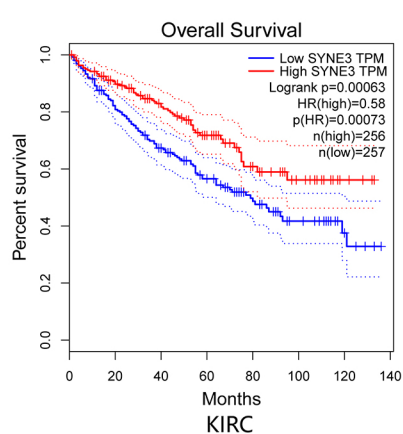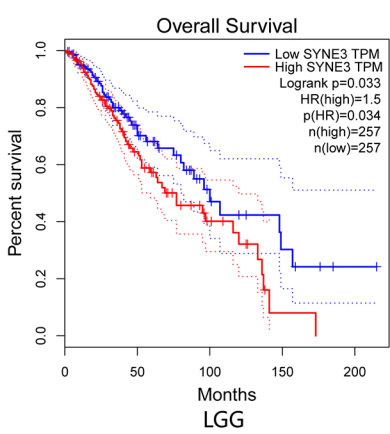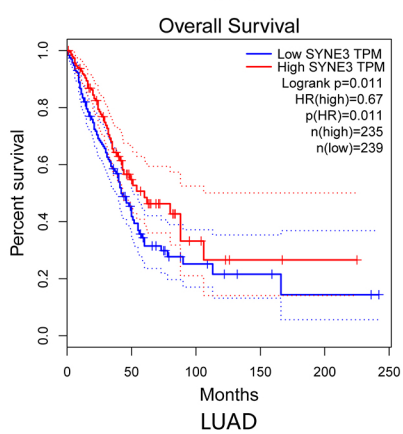

Supplement: Supplementary file 2 — Additional file 2: Figure S2. Prognostic analysis of SYNE3. a Two significant results (KIRC and CESC) of disease free survival analysis on SYNE3, performed on GEPIA. b Five significant results (KIRC, LUAD, CESC, HNSC and LGG) of disease free survival analysis on SYNE3, performed on GEPIA. [file 12967_2020_2521_MOESM2_ESM.pdf]
