# Supplementary material for: Expression profile of SYNE3 and bioinformatic analysis of its prognostic value and functions in tumors
Source: J Transl Med. 2020 Sep 18;18:355. doi: 10.1186/s12967-020-02521-7 (PMC7501639; doi:10.1186/s12967-020-02521-7)
Supplement: Supplementary file 1 — Additional file 1: Figure S1. Expression pattern of SYNE3 refer to database. a Expression profile of SYNE3 in normal tissues acquired from University of California, Santa Cruz (UCSC) database. b SYNE3 RNA Expression in both normal and tumor tissues of human accessed from Gene Expression Profiling Interaction Analysis (GEPIA) database. [file 12967_2020_2521_MOESM1_ESM.pdf]

A

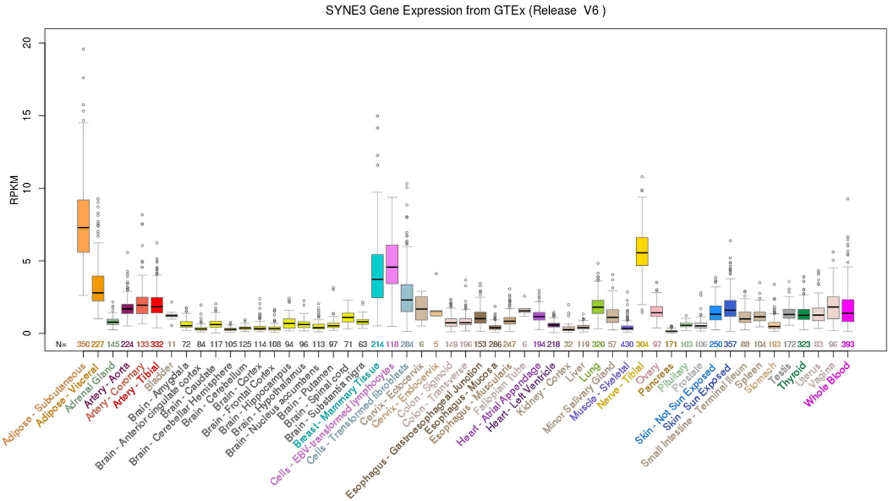
SYNE3 Gene Expression from GTEx (Release V6)
RPKM
20
15
10
5
0
N=
Adipose - Subcutaneous
Adipose - Visceral
Adrenal Gland
Artery - Aorta
Artery - Coronary
Artery - Tibial
Bladder
Brain - Amygdala
Brain - Anterior cingulate cortex
Brain - Caudate
Brain - Cerebellar Hemisphere
Brain - Cerebellum
Brain - Cortex
Brain - Frontal Cortex
Brain - Hippocampus
Brain - Hypothalamus
Brain - Nucleus accumbens
Brain - Putamen
Brain - Spinal cord
Brain - Substantia nigra
Breast - Mammary Tissue
Cells - EBV-transformed lymphocytes
Cells - Transformed fibroblasts
Cervix - Ectocervix
Cervix - Endocervix
Colon - Sigmoid
Colon - Transverse
Esophagus - Gastroesophageal Junction
Esophagus - Mucosa
Esophagus - Muscularis
Fallopian Tube
Heart - Atrial Appendage
Heart - Left Ventricle
Kidney - Cortex
Liver
Lung
Minor Salivary Gland
Muscle - Skeletal
Nerve - Tibial
Ovary
Pancreas
Pituitary
Prostate
Skin - Not Sun Exposed
Skin - Sun Exposed
Small Intestine - Terminal Ileum
Spleen
Stomach
Testis
Thyroid
Uterus
Vagina
Whole Blood

B

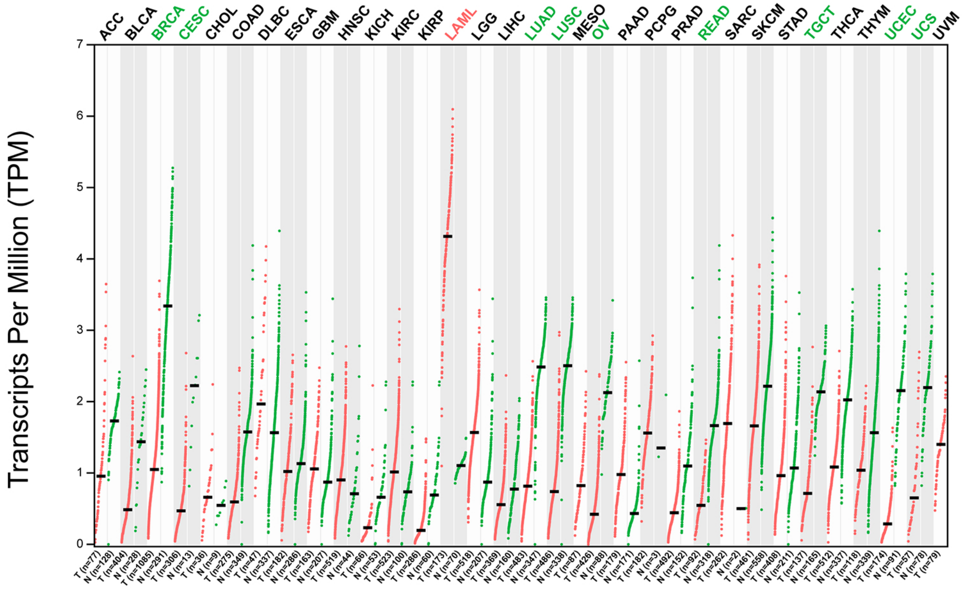
ACC BLCA BRCA CESC CHOL COAD DLBC ESCA GBM HNSC KICH KIRC KIRP LAML LGG LIHC LUAD LUSC MESO OV PAAD PCPG PRAD READ SARC SKCM STAD TGCT THCA THYM UCEC UCS UVM
Transcripts Per Million (TPM)
7
6
5
4
3
2
1
0
